# A pilot randomized trial of conventional versus advanced pelvic floor exercises to treat urinary incontinence after radical prostatectomy: a study protocol

**DOI:** 10.1186/s12894-015-0088-4

**Published:** 2015-09-16

**Authors:** Daniel Santa Mina, Darren Au, Shabbir M. H. Alibhai, Leah Jamnicky, Nelly Faghani, William J. Hilton, Leslie E. Stefanyk, Paul Ritvo, Jennifer Jones, Dean Elterman, Neil E. Fleshner, Antonio Finelli, Rajiv K. Singal, John Trachtenberg, Andrew G. Matthew

**Affiliations:** University Health Network, Toronto, Ontario, 200 Elizabeth Street, Toronto, Ontario M5G 2C4 Canada; University of Guelph-Humber, 207 Humber College Boulevard, Toronto, Ontario M9W 5L7 Canada; University of Toronto, 27 King’s College Circle, Toronto, Ontario M5S 2W6 Canada; University of Guelph, 50 Stone Rd E, Guelph, Ontario N1G 2W1 Canada; Pelvic Health Solutions, 372 Hollandview Trail, Aurora, Ontario L4G 0A5 Canada; York University, 4700 Keele St, Toronto, Ontario M3J 1P3 Canada; Cancer Care Ontario, Toronto, Ontario Canada; Toronto East General Hospital, Toronto, Ontario M4C 5T2 Canada

## Abstract

**Background:**

Radical prostatectomy is the most common and effective treatment for localized prostate cancer. Unfortunately, radical prostatectomy is associated with urinary incontinence and has a significant negative impact on quality of life. Pelvic floor exercises are the most common non-invasive management strategy for urinary incontinence following radical prostatectomy; however, studies provide inconsistent findings regarding their efficacy. One potential reason for sub-optimal efficacy of these interventions is the under-utilization of regional muscles that normally co-activate with the pelvic floor, such as the transverse abdominis, rectus abdominis, and the diaphragm. Two novel approaches to improve urinary continence recovery are ‘Pfilates’ and ‘Hypopressives’ that combine traditional pelvic floor exercises with the activation of additional supportive muscles. Our study will compare an advanced pelvic floor exercise training program that includes Pfilates and Hypopressives, to a conventional pelvic floor exercises regimen for the treatment of post-radical prostatectomy urinary incontinence.

**Methods/Design:**

This is a pilot, randomized controlled trial of advanced pelvic floor muscle training versus conventional pelvic floor exercises for men with localized prostate cancer undergoing radical prostatectomy. Eighty-eight men who will be undergoing radical prostatectomy at hospitals in Toronto, Canada will be recruited. Eligible participants must not have undergone androgen deprivation therapy and/or radiation therapy. Participants will be randomized 1:1 to receive 26 weeks of the advanced or conventional pelvic floor exercise programs. Each program will be progressive and have comparable exercise volume. The primary outcomes are related to feasibility for a large, adequately powered randomized controlled trial to determine efficacy for the treatment of urinary incontinence. Feasibility will be assessed via recruitment success, participant retention, outcome capture, intervention adherence, and prevalence of adverse events. Secondary outcomes of intervention efficacy include measures of pelvic floor strength, urinary incontinence, erectile function, and quality of life. Secondary outcome measures will be collected prior to surgery (baseline), and at 2, 6, 12, 26-weeks post-operatively.

**Discussion:**

Pfilates and Hypopressives are novel approaches to optimizing urinary function after radical prostatectomy. This trial will provide the foundation of data for future, large-scale trials to definitively describe the effect of these advanced pelvic floor exercise modalities compared to conventional pelvic floor exercise regimes for men with prostate cancer undergoing radical prostatectomy

**Trial registration:**

Clinicalstrials.gov Identifier: NCT02233608.

## Background

Radical prostatectomy (RP) is the most common treatment for localized prostate cancer (PCa) [1, 2] with a >90 % 15-year disease-specific survival for men with localized disease [3]. Unfortunately, RP is associated with post-operative urinary incontinence (UI) that can persist for two years or longer and is related to significant reductions in overall health-related quality of life (QoL) [4–7]. Moreover, UI can be an important economic burden to patients due to the cost of pads and lost work productivity [8]. Given the prevalence of RP in the management of PCa and the associated psychosocial, functional, and economic adversity caused by UI, expediting the recovery of urinary control is a major priority for patients and their clinicians.

**Fig. 1:**
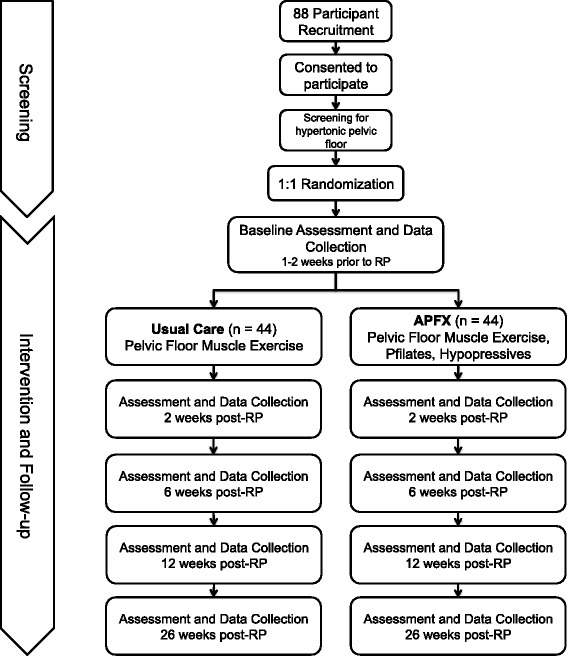
Participant Flow

Normally, the pelvic floor muscles (comprised of the internal sphincter, levator ani, coccygeus, striated urogenital sphincter, external anal sphincter, ischiocavernosus, and bulbospongiousus) work in a coordinated fashion to promote urinary control [9, 10]. While the exact etiology of post-RP UI is not well understood, it is hypothesized to result from injury to the internal sphincter and/or an onset of bladder detrusor hyperactivity that can cause urge incontinence through pressure on the bladder walls [10–16]. Consequently, continence becomes dependent on the pelvic floor musculature that supports the *external* urethral sphincter [12, 17, 18], and thus voluntary conditioning of these muscles is considered a primary, non-invasive UI management strategy post-RP [19, 20].

Conventional pelvic floor muscle exercises (PFMX) are intended to improve urinary control by increasing the strength, endurance, and coordination of the pelvic floor muscles and functional activation of the external urethral sphincter [21–23]. Moreover, chronic performance of PFMXs is suggested to cause hypertrophy of the periurethral striated muscles, a resultant stiffening and strengthening of the pelvic floor muscles and connective tissues, and an inhibition reflex of the detrusor muscles [24–26]. Collectively, the PFMXs facilitate improved capability for external urethral constriction [9] and relaxed detrusor activity to aid in the recovery of post-RP UI [27, 28]. PFMXs typically involve instructions to ‘lift up’ the pelvic floor to stop the flow of urine [17, 21, 29]. PFMX training starts with pelvic floor muscle identification through biofeedback, typically through active urinary flow control (i.e. voluntarily starting and stopping urination) and cueing (using imagery to identify and activate the correct muscles). Once appropriate control is observed, patients are instructed to practice the same contractions routinely with a target volume, intensity, frequency, and/or duration [23].

A recent Cochrane review on the management of UI after RP found a small to moderate benefit of conventional PFMXs; however, none of the included studies incorporated training of the surrounding muscles [21]. The paucity of evidence and modest benefits related to the management of UI with the engagement of surrounding muscle to the pelvic floor is salient because of the growing literature demonstrating that pelvic floor muscle contraction is optimized with co-activation of the abdominals and other regional muscles [30–34]. In particular, the transverse abdominis (TrA), rectus abdominis, and diaphragm muscles are often neglected in PMFX approaches despite their requirement for optimal pelvic floor activation [30, 33]. The relationship between the TrA and diaphragm with pelvic floor activation is described in several lines of research. First, Neumann et al. [33] observed that relaxation of the abdominal wall during pelvic floor muscle contraction only elicits 25 % of the maximal voluntary contraction of the pelvic floor. Second, research has indicated that the likelihood of poor pelvic floor tonic activity (autonomic contraction), and consequent risk of UI, is apparent when the ability of the TrA to maintain a contraction is impaired [30–32]. Third, improved tonic activity of the pelvic floor may improve the autonomic urethral constriction that could prevent leakage without conscious intervention [35, 36]. Similarly, ‘deep belly’ breathing exercises that emphasize diaphragmatic contraction and relaxation have been shown to improve pelvic floor muscle activation and reduce intra-abdominal pressure in women with incontinence [30, 37, 38].

More recent PFMX paradigms incorporate techniques aimed at optimizing pelvic floor muscle responsiveness and contraction quality through the utilization of other regional muscles. One such approach is “Pfilates” (‘*P*elvic *F*loor P*ilates*’) that incorporates the fundamental elements of Pilates (a form of exercise that focuses on core strength, stability, flexibility, and muscle control, as well as posture and breathing) [39, 40] with targeted pelvic floor activation [41]. Pfilates includes several static poses that activate the TrA, hip adductors, gluteal, and pelvic floor muscles with instructions to pulse (small range of motion) with short, maximal effort contractions of the engaged muscles. A recent study by Culligan et al. [40] demonstrated comparable improvements in pelvic floor strength measured by perineometry after 12 weeks of conventional PFMX versus Pilates in 62 women with little or no pelvic floor muscle dysfunction. Although UI was not measured, this study suggests that Pilates may produce similar benefits for UI as traditional PFMXs.

Another novel approach to PFMXs is known as ‘Hypopressive’ exercises. Hypopressive exercises emphasize engaging the TrA with conscious coordination of the diaphragm with breathing that is hypothesized to increase muscle tone of the pelvic floor muscles and subsequently cause urethral constriction [30, 42–45]. While executing the prescribed Hypopressive techniques, the use of deep breathing followed by a brief breath-hold causes relaxation of the diaphragm, decrease in intra-abdominal pressure, and a reflex contraction of the pelvic floor muscles, unconsciously maximizing a contraction and consequently improving re-conditioning of these muscles [43, 45]. Caufriez [46] communicated this technique in 1997 and described the steps as follows: slow diaphragmatic inspiration, followed by total expiration and, after glottal closure, a gradual contraction of the abdominal wall muscles, with superior displacement of the diaphragm cupola (referred to as diaphragmatic aspiration). There is significant attention drawn to the distension of the ribs, breathing, and body positions, so that even though one is aware of their pelvic floor, it is an unconscious movement [30, 38, 43]. Early research on the effects of Hypopressive exercise programs have demonstrated increased tonic activity, strength, and size of the pelvic floor muscles via ultrasonography imaging [44].

To date, no study has assessed the effect of a comprehensive PFMX regimen that includes Pfilates and Hypopressives for UI. This represents a major gap in our understanding of non-invasive UI management strategies for PCa patients especially since the benefits of PFMX are modest and emerging literature suggests that the pelvic floor muscles are suboptimally activated during more focused pelvic floor training.

## Methods

This study is a 2-arm, pilot randomized controlled trial (RCT) that compares the effect of a conventional PFMX program, considered usual care (UC), to an advanced pelvic floor exercise program (APFX) including Pfilates, and Hypopressive for the management of UI after RP for PCa. The primary objective of this study is to assess the feasibility of conducting a full-scale RCT of similar design. Feasibility will be determined via recruitment success, participant retention, outcome capture, intervention adherence, and prevalence of adverse events. Our secondary objectives are to compare the efficacy of APFX to UC in UI, pelvic floor strength, erectile function, and QoL after RP. This study will be conducted at the Wellness and Exercise for Cancer Survivors (WE-Can) program at the Princess Margaret Cancer Centre. WE-Can is a multidisciplinary team of physicians, physiotherapists, kinesiologists, and other exercise professionals that provide exercise and physical activity programming for cancer survivors.

This study has been approved by the ethics boards of the participating institutions and all participants will be asked to provide voluntary and informed consent.

### Study population/participants

Eighty-eight participants will be recruited for this trial. We anticipate an attrition rate of 20 % by the 26-week follow-up assessment to yield 70 participants (35 participants per group) for main efficacy estimate analyses that is aligned with recommended pilot and feasibility study sample sizes for treatment effect estimates [47–49]. *Inclusion Criteria*: Patients that: i) have localized PCa who have consented for RP (open retropubic, laparoscopic, or robot-assisted laparoscopic); ii) are between the ages of 40 and 80 years; and iii) are proficient in English. *Exclusion Criteria*: Patients that: i) are diagnosed with a known neurological disease, autoimmune connective tissue disorder; ii) have prior experience with pelvic floor training by a healthcare provider; iii) have uncontrolled hypertension; iv) have diagnosed chronic obstructive pulmonary disease (COPD) and/or chronic restrictive respiratory disease; v) have a history of inguinal herniation; or vi) have hypertonic pelvic floor muscles upon baseline evaluation. Pelvic floor tonicity will be assessed by a specially trained pelvic floor physiotherapist via digital rectal examination. Hypertonic pelvic floor is determined by the physical examination findings of extrapelvic musculoskeletal and connective tissue examination, as well as the elements of patient history [50]. These patients who exhibit levator ani hypertonicity (tension myalgia) will be excluded as they can experience pelvic, urogenital, and rectal pain; tightness and spasticity; and adverse effects on sexual, urinary, and bowel function that may be exacerbated with contraction-based pelvic floor training [51, 52].

### Study recruitment and randomization

Recruitment will occur in the Greater Toronto Area (total population 4.5 million) in urological oncology clinics and through presentations and/or information stands [52] set-up at local PCa support group meetings. These peer-support meetings often include men who have been recently diagnosed and are considering various treatments for PCa, including RP. Men who self-identify as eligible will receive further verbal and written information about the study. Those deemed eligible and are interested will be asked to provide written consent.

Participants will be 1:1 randomized to the UC and APFX groups, stratified by age (±60 years). Blinded allocation of the participants to their treatment groups will be performed via a process consisting of placing the intervention assignments into opaque envelopes, sealing and shuffling the envelopes, and sequentially numbering them with each new participant receiving an envelope in sequence of recruitment. The envelopes will be opened by the research coordinator with the participant following the baseline assessment, prior to RP Fig. 1.

## Study arms

The two groups will begin participation in their respective interventions immediately following post-operative catheter removal until 26-weeks post-operatively. The physiotherapist assessment of pelvic floor muscle activity and strength will be assessed during each visit in order to optimize the quality of the contractions. Instructions to correctly contract their pelvic floor will be provided verbally and with biofeedback via the Modified Oxford Scale (MOS). Grading of this scale is described below. The research coordinator, who is a Registered Kinesiologist with training and experience in pelvic floor strengthening, Pfilates, and Hypopressives, will be providing the intervention prescription for both study arms and will communicate weekly with the participants to support and quantify program compliance (# of completed contractions/# prescribed contractions), facilitate prescribed progression, and address any barriers to program participation. Furthermore, both groups will receive a manual that includes detailed information and instructions relevant to their training regimens (Tables 1 and 2).

**Table 1:** UC, conventional pelvic floor muscle exercise prescription

Week	Positions	Reps each position	Sets	Contractions daily
1 & 2	Lying and sitting	15-20	2-3	30-60
3 & 4	Lying, sitting, and standing	30-40	2-3	60-120
5 & 6		40-50	3	120-150
7 – 26		50-60	3	150-180

**Table 2:** Advanced pelvic floor muscle exercise prescription

Week	Exercise	Position/pose	Reps	Sets	Hold (sec)	Pulses (Pfilates only)
1	Kegels	Lying and sitting	10-12	3	5-10	
	Pfilates	Butterfly	5	3	3-5	5
	Hypopressive	Diaphragmatic breathing (lying)	3	3	Fully inhale and exhale	
2	Kegels	Lying and sitting	10-12	3	5-10	
	Pfilates	Butterfly	5	2	5-8	5
		Bridge	5	2	3-5	5
	Hypopressive	Standing	3	3	5-10	
3	Kegels	Lying, sitting, and standing	10-12	2	8-10	
	Pfilates	Butterfly	5-10	2	8-10	5-10
		Bridge	5-10	2	5-8	5-10
		Lunge	5-10	2	3-5	5-10
	Hypopressive	Standing	3	2	10-15	
		Kneeling	3	2	5-10	
4	Kegels	Lying, sitting, standing	10-12	3	8-10	
	Pfilates	Butterfly	8-10	2	10	8-10
		Bridge	8-10	2	10	8-10
		Lunge	8-10	2	5-8	8-10
	Hypopressive	Standing	3	1	10-20	
		Kneeling	3	1	10-15	
		Downward kneel	3	1	10-15	
5	Kegels	Lying, sitting, standing	10-12	3	8-10	
	Pfilates	Butterfly	10-12	2	10	10-12
		Bridge	10-12	2	10	10-12
		Lunge	10-12	2	10	10-12
	Hypopressive	Standing	3	1	10-30	
		Kneeling	3	1	10-20	
		Downward Kneel	3	1	10-20	
		Sitting	3	1	10-15	
6-12	Kegels	Lying, sitting, standing	10-12	3	8-10	
	Pfilates	Butterfly	10-12	3	10	10-12
		Bridge	10-12	3	10	10-12
		Lunge	10-12	3	10	10-12
	Hypopressive	Standing	3	1	10-30	
		Kneeling	3	1	10-30	
		Downward Kneel	3	1	10-30	
		Sitting	3	1	10-30	
12-24	Kegels	Lying, sitting and standing	10-15	3	8-10	
	Pfilates	Butterfly	10-15	3	10	10-15
		Bridge	10-15	3	10	10-15
		Lunge	10-15	3	10	10-15
	Hypopressive	Standing	3	1	10-30	
		Kneeling	3	1	10-30	
		Downward Kneel	3	1	10-30	
		Sitting	3	1	10-30	

### Usual care

The UC group will receive generic PFMX exercise instructions and demonstrations from the research coordinator at the initial post-operative time point (post-operative day #10-14) comparable to standard practice for RP patients at the study site. Participants will be instructed on how to contract the pelvic floor using verbal cues and biofeedback (self-observation of urinary control at the toilet). After the pelvic floor muscles have been identified, the UC prescription will consist of maximal voluntary contractions with escalating repetition volume every 2 weeks. Repetition volume will start at 30–60 repetitions per day during weeks 1–2; 60-120/day during weeks 3–4; and 120-150/day during weeks 5–6, and 150-180/day for weeks 7–26. Total daily contractions will be divided into multiple sets over the course of the day, aiming for 10–20 repetitions per set. The total number of repetitions will be divided equally between rhythmic (contract and relaxed over one second) and sustained contractions (contract and hold for up to 10s and relax). Table 1 provides a detailed description of the UC PFMX prescription and progression.

### Advanced Pelvic Floor Exercise (APFX)

Participants will start with the introduction of basic PFMX (comparable to UC) with a gradual integration of Pfilates and Hypopressive exercises until week 12. The Pfilates exercises will progressively integrate postures that engage and activate supportive abdominal muscles including the TrA, hip adductors, and gluteals. During each pose, participants will be asked to perform a series of pulses within a small range of motion while maximally contracting their pelvic floor simultaneously. Similarly, the Hypopressive techniques are gradually integrated in the prescription and progressed over 12 weeks. These exercises focus on diaphragmatic breathing and TrA activation in various static postures. Participants are instructed to perform three successive slow diaphragmatic inspirations, followed by a total expiration and apnea (breath hold). Each apnea will be performed for approximately 10–30 seconds while activating their TrA and intercostal muscles and rising of their hemidiaphragm [45]. APFX participants will be gradually progressed in postures and repetition volume, varying between PFMX, Pfilates, and Hypopressives. Weeks 13–26 comprise the maintenance stage where patients will maintain their prescription until the end of the trial. A detailed week-by-week description of the program is provided in Table 2 and Appendix A. Total APFX contraction volume reflects the volume prescribed in the UC group following post-operative weeks.

## Feasibility assessment

Prior pelvic floor training trials in PCa patients undergoing RP have observed recruitment rates of 21-70 % [21]. We will measure recruitment success through participant recruitment per week and record reasons for non-participation from those who inquire about the study and are eligible to participate but refuse. Adherence to the UC and APFX program will be measured through a logbook that is included in their respective manuals as well as a logbook completed by the research coordinator during the weekly telephone communication that will compare prescribed to completed volume of contractions. Retention will be assessed by measuring attrition throughout the intervention period and at each assessment. We will monitor and record non-severe and severe adverse events that occur to participants during the course of this study using the National Cancer Institute Common Terminology Criteria for Adverse Events version 4.0 [53].

## Outcome measures

Participants will complete five study assessments: baseline (approximately 1 week prior to RP), and at 2, 6, 12, 26-weeks post-operatively. Each assessment session will take place in Toronto, Ontario at the Princess Margaret Cancer Centre in the WE-Can Program. Participant demographics and self-reported disease and treatment-related variables will be collected at the baseline assessment.

### Urinary incontinence

UI will be assessed using the 24-hour pad test, a 3-day, bladder diary, and a single-item on the Patient-Oriented Prostate Utility Scale (PORPUS). The 24-hour pad test will be used to measure UI by assessing the quantity of urine lost in one day. A urinary leakage pad is measured after a 24-hour period and compared to the unused pad weight and is used to assess the severity of UI [54–56]. Patients will receive their pre-weighed pads (TENA® Men’s Protective Guards [57]) at baseline with accompanying plastic zipper storage bags which will be collected at their scheduled assessments. Additional pads per assessment will be provided in case of severe leakage. The pads will be individually weighed on an Ohaus® SP2001 (Ontario, Canada) scale, accurate to +/− 0.1 g. Continence is defined as a loss of ≤ 2 g of urine or the use of one or less pad per day [56, 58–60]. During the 24-hour period that the pad is worn, the participants will complete a frequency volume chart including urination frequency, times of UI, and if the pad was ever removed for a period of time. The 3-day bladder diary is a standard instrument for self-reporting voiding patterns. Items include fluid intake, frequency of toilet voids, episode of urine loss, nocturia, number of pads used, and activity during event for the three-day period. Bladder diaries are widely used in clinical trials assessing UI after prostatectomy [18, 29, 61–63]. Participants will be instructed to complete these 3 days prior to their scheduled assessment appointments. Finally, a single item regarding urinary leakage and bladder control is selected from the PORPUS as an additional self-reported measure of Post-RP UI that has been used in previous studies [64, 65].

### Pelvic floor muscle strength

Digital rectal examination of the pelvic floor by a specially trained pelvic floor physiotherapist is currently the standard clinical method for assessing pelvic floor strength and function [9, 17, 66]. Pelvic floor strength will be performed in the crook lying position and participants are instructed to lift and squeeze the pelvic floor muscles as strongly as possible for a maximum of 5 seconds. The best of 3 maximum contractions is recorded. The quality of contractions are graded using the 6-point Modified Oxford Scale (MOS) [67-69]: 0 = no discernible PFM contraction; 1 = flicker, or pulsing under the examining finger, a very weak contraction; 2 = a weak contraction, an increase in tension in the muscle without any discernible lift or squeeze; 3 = a moderate contraction characterized by a degree of lifting of the posterior pelvic wall and squeezing on the base of the finger with in-drawing of the perineum; 4 = a good PFM contraction producing elevation of the posterior pelvic wall against resistance and in-drawing of the perineum; 5 = a strong contraction of the PFM; strong resistance can be given against elevation of the posterior pelvic wall. This will be measured at baseline (approximately 1 week prior to surgery), and post-operatively at 6, 12, 26-weeks. The MOS has been used in multiple studies assessing pelvic floor muscle strength following RP [56, 59, 70, 71].

### Body composition

Research has shown that men who are overweight reported lower post-operative urinary function [72, 73]. Body mass index (kg/m^2^) will be calculated using participant’s height (m) and weight (kg). Body fat percentage will be assessed by bioelectrical impedance analysis (Tanita© 3000A, Tokyo, Japan). Waist to hip circumference ratio will be measured according to the World Health Organization protocol [74]. Waist circumference will be measured with the measuring tape positioned at the midpoint between lowest margin of the last palpable rib and the top of the iliac crest and hip circumference will be measured at the widest girth of the gluteal region.

### Quality of life

PCa-specific QOL will be measured using two widely used and psychometrically valid and reliable measures: the Functional Assessment of Cancer Treatment-Prostate (FACT-P) [75] and the PORPUS [75-78]. Additional urological symptoms are assessed using the valid and reliable, 7-item International Prostate Symptom Score (IPSS) [79, 80]. Erectile function is assessed using the 5-item International Index of Erectile Function (IIEF) scale, a widely used, psychometrically validated multidimensional self-report instrument evaluating male sexual function [81, 82].

### Physical activity

Recreational physical activity volume will be measured through the reliable and valid 3-item Godin Leisure-Time Exercise Questionnaire – Leisure Score Index (GLTEQ-LSI) [83, 84] . The GLTEQ-LSI assesses the frequency of mild, moderate, and strenuous bouts of leisure physical activity or exercise performed for at least 15 minutes over the past week and has been previously used in trials with PCa survivors [65, 85].

## Statistical analysis

Participant characteristics at baseline will be compared using independent sample t-tests or chi-square tests. We will report retention and compliance rates for the sample and their associated 95 % CI as well as reasons for non-participation of eligible patients. To calculate outcome capture, we will calculate the proportion of participants who have complete data on each outcome at each time point divided by the total number of study participants. Estimates of efficacy (Group and Time main effect, as well as Group x Time interactions) will be analyzed using a repeated-measure analysis of covariance (ANCOVA), controlling for the baseline value of the outcome of interest. We will examine the effect size (Cohen’s d) of the intervention on the clinical outcomes by dividing the observed mean between-group difference in change in the outcome measure from baseline to follow-up (UC vs. APFX) by its standard deviation.

## Discussion

PFMX continues to be the mainstay for conservative management of UI after RP; however, its efficacy is modest. One hypothesis for ineffective PFMX regimens is that the pelvic floor muscles are not adequately activated because ancillary pelvic and abdominal muscles are not concurrently engaged. Novel techniques to address UI secondary to pelvic floor muscle weakness and/or RP involve a more comprehensive strengthening of the pelvic floor and its surrounding structures. Studies have reported a higher maximal voluntary contraction of the pelvic floor when training the pelvic floor muscles and its surrounding muscles simultaneously (i.e. TrA, hip adductors, gluteal, diaphragm) [30–34]. These advanced pelvic floor training techniques include Pfilates and Hypopressives that also engage the TrA, rectus abdominis, gluteals, diaphragm, and hip abductors that enhance pelvic floor contraction. Synergistic training of abdominal and pelvic floor muscles presents a newer approach to the rehabilitation of pelvic floor dysfunction following RP.

The primary outcome of this pilot study is to determine the feasibility of conducting a full-scale RCT of a comprehensive pelvic floor conditioning program compared to conventional PFMX. Measures of outcome efficacy will also be measured in urinary function, pelvic floor muscle strength, and QoL. To our knowledge, this is the first study to examine the effect of a comprehensive PFMX regimen that includes Pfilates and Hypopressives for UI. The above-mentioned training techniques may prove to be an effective alternative in the conservative management of UI and the early recovery of continence after RP.

## Appendix A. Sample cueing instructions for Pfilates and Hypopressives exercises

### Pfilates exercises

Pfilates exercise incorporates a series of poses and plyometric exercises to compliment pelvic floor muscle training. This form of neuromuscular conditioning is a therapeutic alternative for basic pelvic floor muscle exercise that can be easily integrated into any regular exercise routine. In these exercises, the engagement of the external hip rotators, adductors of the thigh, transversus abdominis, and gluteal muscles will facilitate or induce pelvic floor activation.

For each Pfilates pose will follow a series of identical phases:

I. The positioning/movement: Here you will position yourself in the Pfilates pose and perform the movement a series of repetitions
II. The hold: during this phase you engage the active muscles and contract your pelvic floor muscles
III. The pulse (plyometric): In this last phase, you will perform a series of pulses, matching the number of repetitions in the first phase. (i.e. If you performed 10 repetitions in the movement phase, you will perform another 10 repetitions in the pulse phase).

### Hypopressive exercises

Hypopressives are performed mainly via the transversus abdominis activation. The goal of these exercises is to relax the diaphragm, which in turn decreases the intra-abdominal pressure and may activate the abdominal and pelvic floor muscles simultaneously.

Each pose will follow a series of identical phases:

I. Positioning of the pose
II. Three diaphragmatic breaths (rest breaths), described below
III. Apnea (breath hold) of 5-30 seconds
IV. Repeat these steps a total of three times for each pose

### Diaphragmatic breathing (rest breaths) – practice pose

1. Lie on your back on a flat surface with your knees bent and place your hands on the bottom of your ribs.
2. Breathe in slowly through your nose and open your upper chest as much as possible. You should feel your chest flare out ward with your hands.
3. Exhale fully through pursed lips.

### Apnea

To perform a correct apnea (breath hold) you must close your nose and mouth (glottal stop). When you are performing the apnea phase of the exercise, you will have fully **exhaled** from your last rest breath. At this point, with a glottal stop, perform an inhalation without taking air in. You should feel your stomach and abdomen lift and squeeze up to your ribcage. Each apnea is held for 10 to 30 seconds.

## Abbreviations

ANCOVA: Analysis of covariance
APFX: Advanced pelvic floor exercise
FACT-P: Functional assessment of cancer therapy-prostate
GLTEQ-LSI: Godin leisure time exercise questionnaire – leisure score index
IIEF: International index of erectile function
IPSS: International prostate symptom score
MOS: Modified Oxford scale
PCa: Prostate cancer
PFMX: Pelvic floor muscle exercise
PORPUS: Patient-oriented prostate utility scale
QOL: Quality of life
RP: Radical prostatectomy
TrA: Transverse adominis
UC: Usual care
UI: Urinary incontinence
WE-Can: Wellness and Exercise for Cancer Survivors Program
